# Effects of a lipid-encapsulated zinc oxide dietary supplement, on growth parameters and intestinal morphology in weanling pigs artificially infected with enterotoxigenic *Escherichia coli*

**DOI:** 10.1186/s40781-014-0038-9

**Published:** 2015-01-24

**Authors:** Sung jae Kim, Chang Hoon Kwon, Byung Chul Park, Chul Young Lee, Jeong Hee Han

**Affiliations:** College of Veterinary Medicine and Institute of Veterinary Science, National University, Chuncheon, 200-701 Republic of Korea; R & D Institute, Sunjin Co., Ltd, 517-3 Doonchon-dong, Kangdong-gu, Seoul, 134-060 Republic of Korea; The Regional Animal Industry Center, Gyeongnam National University of Science and Technology, Jinju, 660-758 Republic of Korea

**Keywords:** Diarrhea, Enterotoxigenic *Escherichia coli*, Growth performance, Intestine, Weaning pig, Zinc oxide

## Abstract

The study was performed to investigate the effect of dietary supplementation of a lipid-encapsulated Zinc oxide on growth parameters and intestinal mucosal morphology piglets born to Duroc-sired Landrace × Yorkshire dams. Twenty-four 30-day-old piglets weaned at 25 days of age were orally challenged with 5 × 10^8^ colony forming units of enterotoxigenic *Escherichia coli* (ETEC) K88 and fed one of the four diets for 7 days: (i) a nursery basal diet containing 100-ppm ZnO (referred to as BASAL), (ii) BASAL supplemented with 120-ppm apramycin (referred to as ANTIBIO), (iii) BASAL with 2,400-ppm ZnO (referred to as HIGH), and BASAL containing 100-ppm lipid-encapsulated ZnO (referred to as LE). All piglets were killed at the end of the experiment for histological examination on the intestine. The results showed that the average daily gain (ADG), the villus height: crypt depth (CD) ratio in the ileum, and the goblet cell density of the villus and crypt in the duodenum, jejunum, and colon were greater in the LE-fed group that those of the BASAL (p < 0.05). Fecal consistency score (FCS) and the CD ratio in the ileum were less in the LE-fed group, compared to the BASAL-fed one (p < 0.05). The effects observed in the LE-fed group were almost equal to those of the HIGH-fed group as well as even superior to those of the ANTIBIO-fed group. Taken together, our results imply that dietary supplementation of 100-ppm lipid-encapsulated ZnO is as effective as that of 2,400-ppm ZnO for promoting growth diarrhea and intestinal morphology caused by ETEC infection.

## Background

Dietary supplementation of 2,000- to 3,000-ppm of zinc oxide (ZnO) alleviates diarrhea and improves growth performance and the integrity of intestinal morphology of post-weaning piglets [1-4]. Moreover, dietary ZnO at those pharmacological doss exhibits the similar effects in the enterotoxigenic *Escherichia coli* (ETEC)-challenged piglets, which have been used as a model host for post-weaning diarrhea caused by ETEC infection [5-7].

A lipid-encapsulated proprietary Zn supplement whose active component ZnO is supposedly released after digestion of the lipid coating by lipase in the intestinal lumen. The ZnO particle of the mineral supplement is more efficiently delivered to the intestine than native ZnO because ZnO contained in the former is not ionized in the stomach owing to the lipid coating [8].

We have previously reported that dietary supplementation of 100-ppm of the lipid-encapsulated ZnO (LE ZnO) was as effective as 2,500-ppm of native ZnO in alleviating diarrhea and growth retardation of weanling pigs artificially challenged with 3 × 10^10^ colony forming units (CFU) of ETEC K88 [9]. It is questionable, however, whether or not the supplemental LE ZnO would exhibit such effects comparable to those of pharmacological ZnO as well as antibiotics in weanling pigs with infection of ETEC, because the challenge dose of ETEC in our previous study was greater than that necessary to induce a mild clinical infection [5]. The present study was therefore undertaken to determine the effect of dietary supplementation of LE ZnO on growth promotion and intestinal morphology in weanling pigs after infection with a low infectious dose (5 × 10^8^ CFU) of ETEC, compared to that of antibiotics and/or pharmacological concentration of ZnO.

## Methods

### Animals and dietary treatments

The experimental protocol was approved by the Institutional Animal Care and Use Committee of Kangwon National University. Twenty-four castrated male piglets born to Duroc-sired Landrace × Yorkshire dams were randomly allocated to four 2-m^2^ pens at weaning at 25 days of age, with six animals per pen, in the university animal research station. The animals in each pen were adapted to one of the following four experimental diets for 5 days (Table 1), (i) a nursery basal diet containing 100-ppm ZnO (referred to as BASAL), (ii) BASAL supplemented with 120-ppm apramycin (referred to as ANTIBIO), (iii) BASAL with 2,400-ppm ZnO (referred to as HIGH), and BASAL containing 100-ppm LE ZnO instead of 100-ppm ZnO (referred to as LE). On Day 0 of the experiment, each animal received a single oral dose of 5 × 10^8^ cfu of ETEC K88 in 5 mL of PBS (spell out). The animals were provided ad libitum with the prescribed diets and water during the 7-day experimental period after bacterial challenge. The ambient temperature, which was set at 28°C on Day −5, was lowered to 25°C at a rate of 0.5°C/day. The rectal temperature of each piglet was measured on days 0, 1, 4, and 7 post-inoculation. Fecal consistency was scored on days 0, 1, 2, 4, and 7 post-inoculation according to the 3-notch integer scale [10-12]: 1, well-formed feces; 2, sloppy feces; 3, diarrhea. To quantify fecal shedding of ETEC K88, rectal stool samples were collected from each pig on days 1, 4, and 7 post-inoculation.

**Table 1:** **Composition of the basal diet (as-fed basis)**

**Item**	**Content**
Ingredient, %	
ZnO^1^	0.01
Others^2^	999.99
Calculated chemical composition	
Digestible energy, MJ/kg	13.98
Crude protein, %	16.5
Ether extract, %	3.91
Lysine, %	1.13

^1^Substituted by 0.25% uncoated ZnO and 0.01% lipid-encapsulated ZnO in the high-ZnO (‘HIGH’) and lipid-encapsulated ZnO (‘LE’) diets, respectively (See Tables 2, 3, and 4).

^2^Grains-soy-whey-based ingredients, composition of which was reported previously (Kwon et al., [9]). One of the four experimental diets used in the present study contained 120 ppm of apramycin (‘ANTIBIO’).

### Collection of blood samples and intestinal tissues

All the piglets were killed at the end of the feeding trial. Upon opening the abdominal cavity, the intestinal tract was dissected as described previously [9,13]. For morphological examination of the intestinal mucosa, cross-sectional segments (approximately 3 cm in length) were cut approximately at a 10-cm location from the pylorus (duodenum), at 50% (jejunum) and 90% (ileum) of the length of the small intestine, and at the transverse colon. Serum was collected after centrifugation and stored in aliquots at −20°C until used.

### Quantification of fecal shedding of ETEC K88

The bacterial number of ETEC K88 shed in the feces was determined by quantitative real-time PCR targeting a 70-bp fragment of the K88 fimbrial gene as described by West et al. [14]. Briefly, the genomic DNAs in the feces were extracted using QIAamp DNA Mini Kit (Qiagen, Hilden, Germany). The PCR mixture contained 2 μL of DNA template, 100 pmol of the forward primer (5′-GGTTCAGTGAAAGTCAATGCATCT-3′), 100 pmol of the reverse primer (5′-CCCCGTCCGCAGAAGTAAC-3′), 0.5 μL of the probe (Cy5-5′-CCACCTCTCCCTAACACACCGGCAT-3′-BHQ2; GenoTech, Daejeon, Korea), and 12.5 μL of Premix EX Taq DNA polymerase (Takara Bio, Shiga, Japan) in a total volume of 25 μL. Thermal cycling was performed with an initial denaturation at 95°C for 10 min, followed by 45 cycles of 95°C for 20 sec, 62°C for 30 sec, and 72°C for 30 sec. The cfu of ETEC K88 in each fecal sample was assessed from the standard curve for the ETEC K88 standard solution plotted against the threshold (Ct) value using the Smartcycler software (Cepheid, Sunnyvale, CA).

### Histological examination

The cross-sectional segments of the small and large intestines were fixed for 48 hrs in a 10% neutral formalin solution. The fixed tissues were embedded in paraffin and sliced to a thickness of 4 μm, after which the thin sections were mounted on glass slides and stained with hematoxylin/eosin for microscopic examination of the mucosal structure as previously described [13]. For determination of the goblet cell density, the specimens were stained with periodic acid-Schiff and Alcian Blue to count the neutral mucin- and acid mucin-secreting goblet cells, respectively, as described by Uni et al. [15]. The villus height (VH), crypt depth (CD), and the goblet cell number were determined on four well-oriented structures under a 400-fold magnified microscopic field using the Diagnostic Insight visual analysis program (Olympus, Tokyo, Japan) as described previously [9]. In each variable, the average of four measurements was taken as an observation.

### Statistical analysis

Data were analyzed as a completely randomized design using the MIXED procedure of SAS (SAS Inst. Inc., Cary, NC, USA). The model included the dietary treatment as the main effect as well as the day and its interaction with treatment in the analysis of repeated measurements. Effects of the treatment and day including its interaction with treatment were tested using the animal and day × animal nested within treatment as error terms, respectively. Means were separated by the probability difference (PDIFF) option.

## Results

The rectal temperature of the post-weaning pigs increased after the oral challenge with ETEC K88 and remained elevated (*P* < 0.05) during the 7-day experimental period post-challenge (Table 2). The mean temperature of the piglets was lowered by feeding LE when compared with that of the animals provided with BASAL (Table 2). However, the mean rectal temperature of the LE-fed group did not differ from those of the ANTIBIO- and HIGH-fed groups (Table 2).

**Table 2:** **Effects of dietary supplementations of 100 ppm of ZnO without (BASAL) or with 120 ppm of apramycin (ANTIBIO), 2,500 ppm of ZnO (HIGH), and 100 ppm of the lipid-encapsulated ZnO (LE) on clinical signs of post-weaning pigs challenged with 5 × 10** ^**8**^ **cfu of ETEC K88**

**Item**	**BASAL**	**ANTIBIO**	**HIGH**	**LE**	**SEM**	***P-*** **value**
Rectal temperature, °C						
D 0	38.48	38.52	38.50	38.48	0.15^x^	
D 1	39.85^a^	39.47^ab^	39.22^b^	39.57^ab^		
D 4	40.08^a^	39.43^bc^	39.22^c^	39.63^b^		
D 7	39.75^a^	39.32^b^	38.98^bc^	38.85^c^		
Overall^1^	39.54^a^	39.18^b^	38.98^b^	39.13^b^	0.10	<0.01
Fecal consistency score^2^						
D 0	1.50	1.67	1.83	1.50		
D 1	2.83	2.67	2.33	2.50		
D 2	2.67	2.83	2.33	2.50	0.22^x^	
D 4	2.50^a^	2.17^a^	2.17^a^	1.50^b^		
D 7	2.50^a^	1.83^b^	1.67^b^	1.67^b^		
Overall^3^	2.40^a^	2.23^ab^	2.07^b^	1.93^b^	0.11	0.04
Fecal shedding of *E. coli*, Log_10_ cfu/g feces						
D 1	7.00^a^	5.90^b^	5.35^b^	5.58^b^		
D 4	6.86^a^	5.71^b^	5.22^bc^	4.83^c^	0.29^x^	
D 7	7.33^a^	5.50^b^	4.91^bc^	4.25^c^		
Overall^4^	7.06^a^	5.70^b^	5.16^b^	4.89^b^	0.33	<0.01

^1^ *P*-values for the day and day × treatment were < 0.01 and 0.04, respectively.

^2^1, well-formed feces; 2, sloppy feces; 3, diarrhea.

^3^ *P*-values of the day and day × treatment were < 0.01 and 0.25, respectively.

^4^ *P*-values of the day and day × treatment were 0.09 and 0.22, respectively.

^x^Applies to all day × treatment combinations.

^a-c^Means with no common superscript within a row differ (*P* < 0.05).

The FCS was also elevated on Day 1 and 2 above that on Day 0, but returned to the pre-challenge level by d 7 (Table 2). The mean FCS was less in the LE-fed group than in the BASAL- and ANTIBIO-fed groups, but it did not differ between the former and HIGH-fed groups. The mean level of excretion of ETEC K88 to feces expressed as log cfu also was less in the LE-fed group, compared to the BASAL- and ANTIBIO-fed groups, not being different between the former and the HIGH-fed group.

The ADG of the animals was reduced after the ETEC challenge when compared with that during the pre-challenge period (Table 3). The ADG during the experimental period was greater in the LE-fed group than in the BASAL, but it did not differ between the former and either of the ANTIBIO- and HIGH-fed groups.

**Table 3:** **Effects of dietary supplementations of 100 ppm of ZnO without (BASAL) or with 120 ppm of apramycin (ANTIBIO), 2,500 ppm of ZnO (HIGH), and 100 ppm of the lipid-encapsulated ZnO (LE) on growth performance of weanling pigs challenged with 5 × 10** ^**8**^ **cfu of ETEC K88**

**Item**	**BASAL**	**ANTIBIO**	**HIGH**	**LE**	**SEM**	***P-*** **value**
Before the ETEC K88 challenge (d −5 to d 0)^1^						
Initial wt, kg	7.06	6.78	6.91	6.57	0.28	0.65
Final wt, kg	7.88	7.56	7.74	7.40	0.31	0.72
ADG, g	165	156	166	166	11	0.91
After the ETEC K88 challenge (d 0 to d 7)^1^						
Final wt, kg	8.43	8.32	8.66	8.36	0.36	0.91
ADG, g	78^b^	109^ab^	132^a^	137^a^	14	0.03

^1^The ADFI of the animals, which were group-fed, were 236, 233, 224, and 240 g during the pre-challenge period and 324, 386, 350, and 370 g during the post-challenge period in BASAL, ANTIBIO, HIGH, and LE, respectively.

^a,b^Means with no common superscript within a row differ (*P* < 0.05).

The densities of goblet cells in the villus and crypt of the duodenum were greater in the LE-fed group than in the BASAL- and ANTIBIO-fed groups, whereas the villlus and crypt goblet cell densities did not differ between the LE- and HIGH-fed groups (Table 4). The goblet cell density in the villus and crypt of the jejunum was greater in the LE-fed group than in any other group, but in the ileum, it did not differ across the treatments. In the colon, it was greater in the LE-fed group than the BASAL- and ANTIBIO-fed groups, not being different between the LE- and HIGH-fed groups.

**Table 4:** **Effects of dietary supplementations of 100 ppm of ZnO without (BASAL) or with 120 ppm of apramycin (ANTIBIO), 2,500 ppm of ZnO (HIGH), and 100 ppm of the lipid-encapsulated ZnO (LE) on histological measurements of the intestine in weanling pigs challenged with 5 × 10** ^**8**^ **cfu of ETEC K88**

**Item**	**BASAL**	**ANTIBIO**	**HIGH**	**LE**	**SEM**	***P-*** **value**
Goblet cell density, cells/mm^2^						
Duodenum						
Villus	143^b^	145^b^	230^a^	211^a^	13.2	<0.01
Crypt	215^b^	221^b^	377^a^	356^a^	24.3	<0.01
Jejunum						
Villus	102^d^	134^c^	164^b^	192^a^	9.1	<0.01
Crypt	241^c^	260^c^	305^b^	349^a^	11.1	<0.01
Ileum						
Villus	187	196	192	183	18.7	0.97
Crypt	409	403	439	428	15.0	0.32
Colon						
Mucosa	475^b^	515^b^	624^a^	675^a^	32.8	<0.01
Mucosal morphology						
Duodenum						
VH^1^, μm	367	386	424	435	19	0.07
CD^2^, μm	254	252	262	256	11	0.93
VH:CD	1.45	1.53	1.62	1.72	0.08	0.10
Jejunum						
VH, μm	331	340	305	336	17	0.52
CD, μm	257^a^	255^a^	200^b^	205^b^	17	0.04
VH:CD	1.31	1.41	1.52	1.64	0.10	0.12
Ileum						
VH, μm	297	300	295	305	9	0.89
CD, μm	250	239	229	224	7	0.06
VH:CD	1.19^a^	1.26^a^	1.29^ab^	1.36^b^	0.03	0.02

^1^VH = villus height.

^2^CD = crypt depth.

^a-d^Means with no common superscript within a row differ (*P* < 0.05).

The VH, CD and VH:CD ratio in the duodenum were not influenced significantly by the dietary treatment (Table 4). The CD in the jejunum was less in the LE-fed group than in the BASAL- and ANTIBIO-fed groups whereas it did differ between the LE- and HIGH-fed groups. In the ileum, the VH:CD ratio was greater in the LE-fed group than in the BASAL- and ANTIBIO-fed groups, but it did not differ between the former and the HIGH-fed group.

## Discussion

The piglets challenged with 5 × 10^8^ cfu of ETEC K88 exhibited expected responses to both ANTIBIO and HIGH, with decreases in rectal temperature, FCS, and fecal ETEC shedding and increases in ADG and goblet cell density. Moreover, the effects of LE were virtually equal to those of HIGH, which were equal to or greater than those of ANTIBIO. The ADG of the experimental groups were thus reflective of their ETEC infection status, suggesting that the greater weight gain in the LE- and HIGH-fed groups, compared to the BASAL, resulted from the antibacterial activities of the former. Overall, the present results for the ANTIBIO- and HIGH-fed groups were consistent with published reports regarding the effects of apramycin with or without other antibiotics [12,16,17] as well as pharmacological ZnO [1,7,18]. Furthermore, the effects of HIGH and LE-BASAL, compared to BASAL, observed in the present study were very similar to our previous results in post-weaning pigs challenged with 3 × 10^10^ cfu of ETEC K88 [9], suggesting that LE and HIGH are equally effective for alleviating the ETEC infection.

The VH, CD, and VH:CD of the intestinal mucosa of the ETEC-infected piglets did not change in response to HIGH or LE, except for a decrease in CD in the jejunum and an increase in the VH:CD ratio in the ileum. These results were substantially different from the almost consistent increase in VH and VH:CD and decrease in CD in response to both HIGH and LE in the jejunum and ileum in post-weaning pigs challenged with a high dose of ETEC K88 in our previous study [9]. Regarding the effects of supplemental ZnO on intestinal mucosal morphology of the piglets in the literature, the VH and VH:CD increased and CD decreased inconsistently in response to pharmacological ZnO in Li et al. [19,20] and Owusu-Adiedu et al. [18], but in Hedemann et al. [21], the VH decreased due to supplemental ZnO. Collectively, these results suggest that the beneficial effects of supplemental ZnO on the structural integrity of intestinal mucosa are more pronounced when the piglets are severely infected with ETEC than when moderately infected.

## Conclusions

The present results indicated that dietary supplementation of 72 ppm Zn as LE ZnO was as effective as that of 2,000 to 2,500 ppm Zn provided as native ZnO or antibiotics for increasing growth performance, goblet cell density in the intestine as well as reducing diarrhea in weanling pigs challenged with a low dose of ETEC K88. More studies are necessary, however, to determine the effects of LE ZnO relative to those of native ZnO on those responses examined in the present study in naïve weanling pigs under production conditions.
